# Supplementary material for: Repeated Vaginal Exposures to the Common Cosmetic and Household Preservative Methylisothiazolinone Induce Persistent, Mast Cell-Dependent Genital Pain in ND4 Mice
Source: Int J Mol Sci. 2019 Oct 28;20(21):5361. doi: 10.3390/ijms20215361 (PMC6862067; doi:10.3390/ijms20215361)
Supplement: Supplementary file 1 [file ijms-20-05361-s001.pdf]

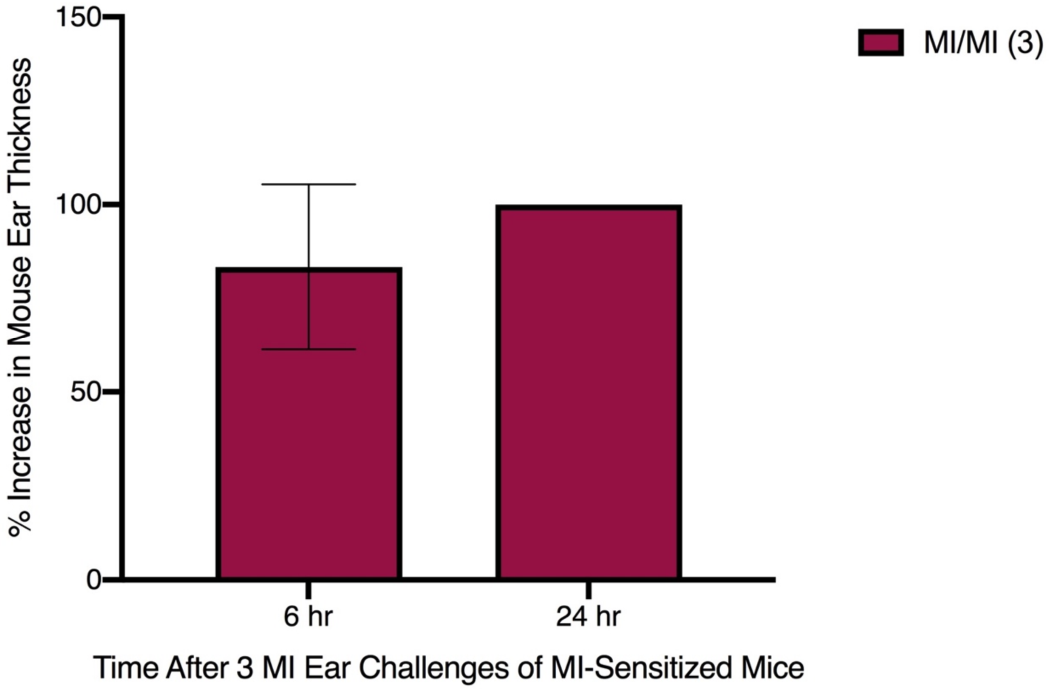

Figure S1. *Ear edema in mice flank-sensitized and subsequently challenged with topical MI application on the ear.* Ear thickness (mm) was measured prior to MI-sensitization and after 3 MI challenges (6 and 24 hours) for each mouse. Represented here is the % increase in mouse ear thickness compared to baseline. (n = 3 mice/treatment group).

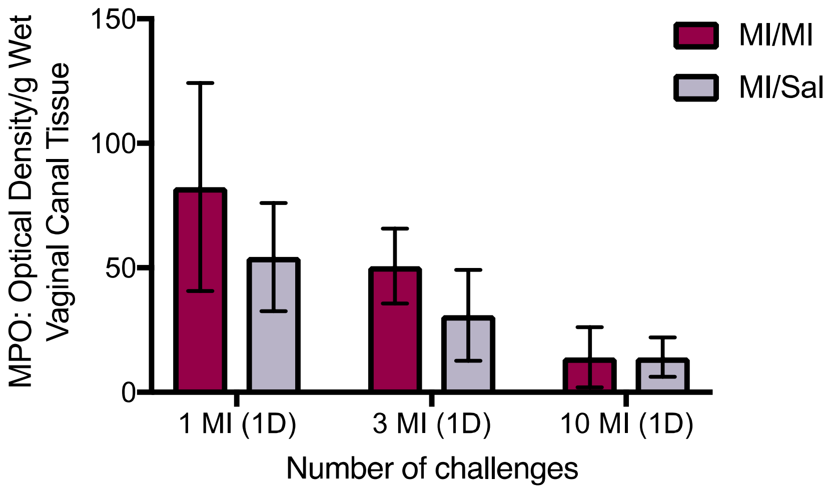

Figure S2. *Myeloperoxidase in the vaginal canal tissue of MI-sensitized mice subsequently challenged with MI or vehicle.* Tissue myeloperoxidase levels measured as optical density/g wet vaginal canal tissue at 1 day after 1, 3 and 10 MI or vehicle challenges, n = 3/treatment group.

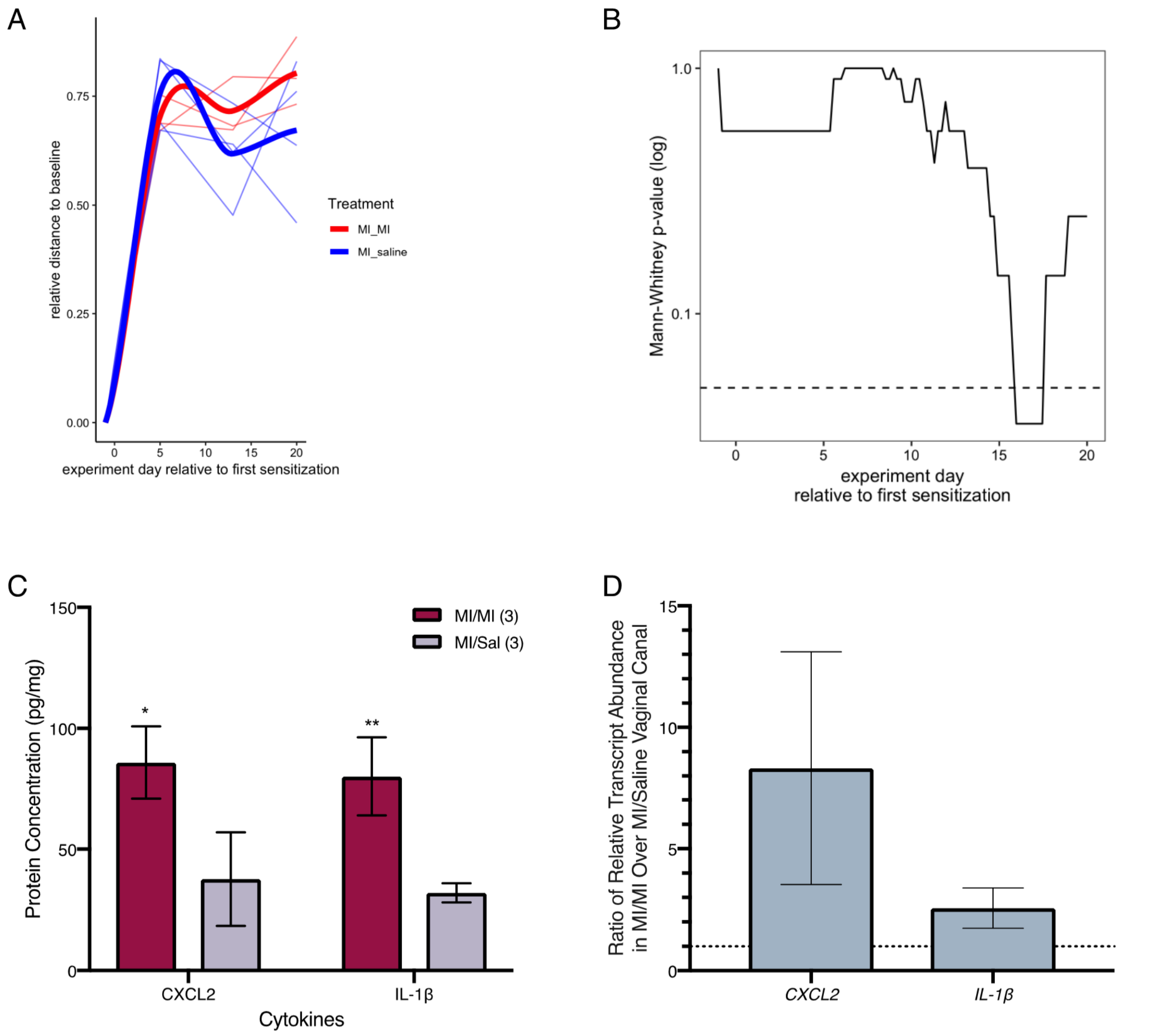

Figure S3. *Destabilized murine vaginal microbiome after 10 intra-vaginal MI challenges in previously sensitized ND4 female mice***.** Mice were sensitized to MI then challenged with MI (red) or saline (blue). Vaginal microbiota was sampled by PBS lavage and the microbial community assessed by 16S rRNA sequencing. To assess microbial community structure stability and recovery, we calculated the unweighted UniFrac distance to pre-sensitization baseline for each timepoint in each animal (**A**). Lighter lines show longitudinal data from individual mice; bold lines show a loess spline fit to the treatment groups' data. (**B**) To test for statistical differences at distinct time points, longitudinal data was modeled using splines and compared using the splinectomeR package in R. **(C)** Protein concentration of CXCL2 and IL-1β 1 day after third challenge in whole cell vaginal canal lysates, determined through ELISA and normalized to total protein concentration in each sample through DC assay. Significance with respect to control group * = $p<0.05$ and ** = $p<0.01$ (n = 5 mice/treatment group). (**D**) Gene expression of *CXCL2* and *IL-1β* in RNA extracted from the vaginal canal 7 days after 10th MI challenge relative to vehicle challenged controls, normalized to *β2-microglobulin* mRNA levels. (n = 5-6 mice/treatment group).
